# A follow-up study on factors affecting the recovery of patients with hypothyroidism in different selenium environments

**DOI:** 10.1186/s12902-024-01536-1

**Published:** 2024-01-29

**Authors:** Si Wang, Ping Chen, Yayi He, Jing Wei, Li Tian, Yajun Wu, Hongjun Lv, Xiaogang Peng, Xingru Zhang, Bingyin Shi, Qian Wu

**Affiliations:** 1 Department of Epidemiology and Biostatistics, School of Public Health, Xi’an Jiaotong University Health Science Center, Xi’an, 710061 Shaanxi China; 2 School of Public Health, Global Health Institute, Xi’an Jiaotong University, Xi’an, China; 3 Endemic Disease Control Institute of Shaanxi Province, Xi’an, Shaanxi, 710003 China; 4 Department of Endocrinology, The First Affiliated Hospital of Xi’an Jiaotong University Health Science Center, Xi’an 710061, China; 5 Lizhou District, Guangyuan Central Hospital, Sichuan Province, Guangyuan City, 628000 China; 6 Xi’an North Hospital, Xi’an, Shaanxi, 710043 China; 7 Ningshan County People’s Hospital, Ningshan, Ankang, 711600 Shaanxi China; 8 Key Laboratory for Disease Prevention and Control and Health Promotion of Shaanxi Province, Xi’an Jiaotong University, Xi’an, China

**Keywords:** Hypothyroidism, AITD, Trace element, Recovery, Micronutrient

## Abstract

**Background:**

Hypothyroidism is a major manifestation of autoimmune thyroid diseases (AITD). We previously reported that a low selenium (Se) status was linked to an elevated prevalence of thyroid diseases. We hypothesized that Se status may also influence the restoration of thyroid function. Thus, this study aimed to investigate the factors affecting the recovery of thyroid function in patients with (sub-)clinical hypothyroidism, with a specific focus on Se status.

**Methods:**

We conducted a 6-year prospective cohort study comparing two counties with different Se concentrations. Demographic and disease data were collected from 1,190 individuals (549 Se-adequate and 641 Se-deficient) who completed a follow-up study in 2019. In addition, urinary iodine (*I*) levels, thyroid function, and serum and nail Se levels were measured. Logistic regression was used to investigate the relationship between Se deficiency and recovery of thyroid function.

**Results:**

Sex and smoking status was similar between the two counties studied. Thyroid function recovery rate was significantly higher in Se-deficient counties (46.0% vs. 30.6%, *P* = 0.008). In the multivariate analysis, our results show that female sex (odds ratio [OR] (95% confidence interval [CI]) = 1.875 (1.080–3.257), *P* = 0.026] and increasing age [OR (95%CI) = 1.028(1.007–1.049), *P* = 0.009] were associated with the recovery rate. Additionally, our study revealed that while Se status was significant in the univariate analysis, this association appeared to disappear in the multivariate analysis.

**Conclusions:**

Female sex and increasing age have unfavorable effects on the recovery of thyroid function in patients over 30 years of age with (sub-) clinical hypothyroidism.

**Supplementary Information:**

The online version contains supplementary material available at 10.1186/s12902-024-01536-1.

## Background

Autoimmune thyroid disease (AITD) is the most common endocrine and autoimmune disease in the general population and is characterized by lymphocyte infiltration of the thyroid gland resulting from a disordered immune system that attacks thyroid follicular cells, leading to hyperthyroidism or hypothyroidism [1–3]. Graves' disease (GD) is characterized by hyperthyroidism, whereas hypothyroidism is the characterized of autoimmune thyroiditis (AIT), with a correlation between autoantibody concentrations and disease severity [4, 5].

The thyroid gland has the highest concentrations of *I* and Se in the human body, both of which are essential for normal thyroid cell function and adequate biosynthesis of thyroid hormones [6, 7]. The concentration of selenium (Se) in the water and plants of a region depends on the Se content of the soil, resulting in varying levels of Se in the blood of individuals living in regions with different Se contents. Observational studies have shown a particular risk of AITD development with insufficient Se intake [8, 9]. However, supplementation studies on AITD have yielded controversial results, especially for AIT, in which positive or neutral effects of Se on thyroid peroxidase antibody titers and thyroid gland structures have been reported [10, 11]. The reason for these inconsistent effects is not yet well understood but may be linked to the baseline Se status [12, 13]. The positive effects of Se supplementation have consistently been observed in patients with GD and are included in clinical practice in countries with borderline Se deficiency [14–18].

In 2013, we conducted a cross-sectional study in two counties in the Shaanxi Province with different soil Se environments. The data from this study indicated that habitually low Se intake and status were associated with an increased risk of developing thyroid diseases, with a particularly high prevalence of AIT [19]. In a subsequent follow-up study conducted over six years, we monitored disease incidence and substantiated these findings by revealing an increased incidence of AIT and a high seroconversion rate of thyroid peroxidase antibody (TPO-Ab) in individuals residing in Se-deficiency areas [20]. However, there is a lack of data regarding the potential link between Se intake and the recovery of thyroid function in patients with AITD. Therefore, we compared patients with AITD in Se-deficient and Se-adequate areas to investigate potential associations.

## Materials and methods

### Study design and participants

**Fig. 1:**
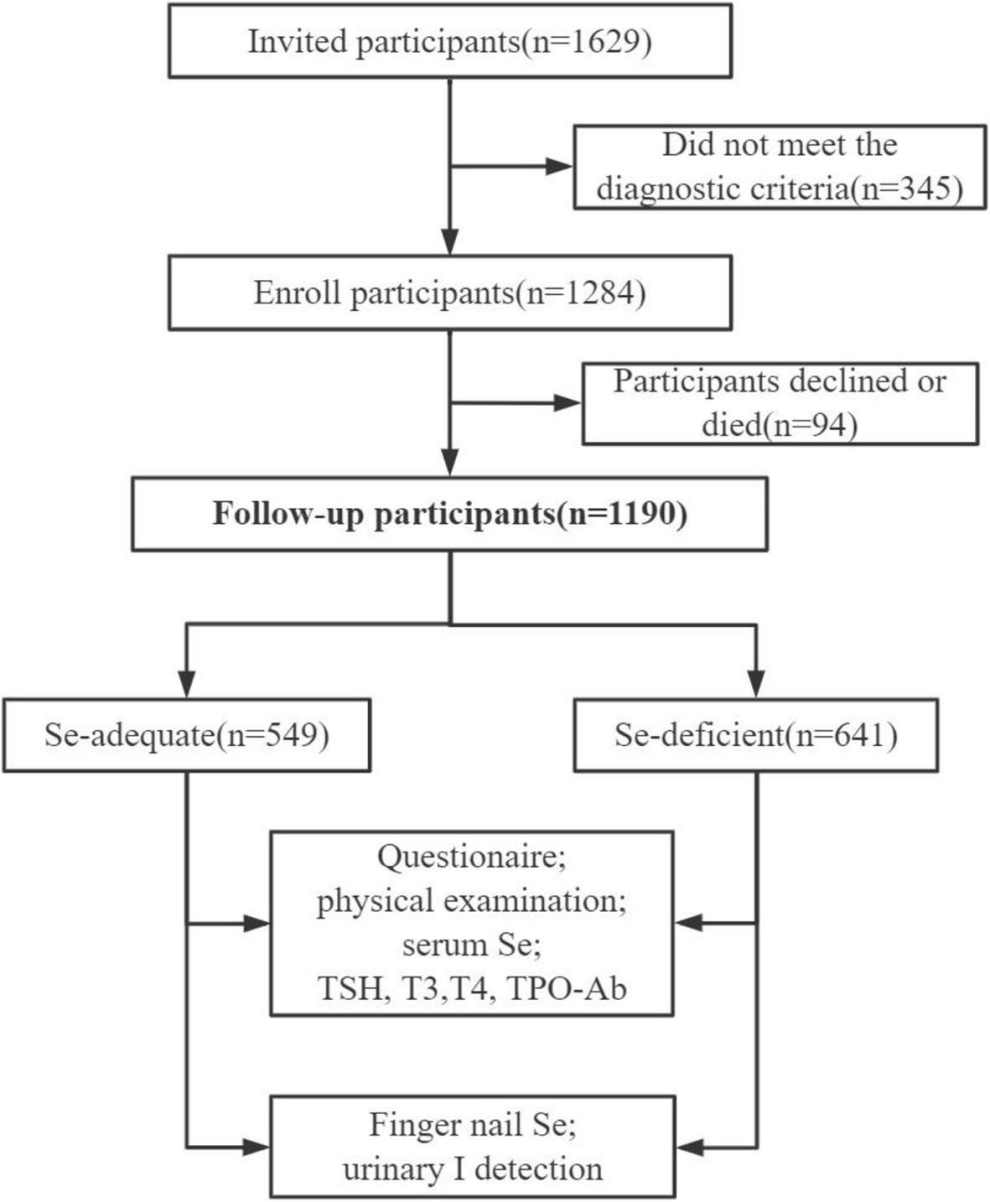
Study process flowchart including number of participants and laboratory analyses

We conducted a six-year prospective observational cohort study following a baseline cross-sectional survey done in 2013 wherein the prevalence of thyroid diseases based on Se status was studied. After six years, participants who had been diagnosed with thyroid-related diseases in the baseline study were reexamined to determine disease progression and thyroid function recovery. Their serum Se, TPO-Ab, and thyrotropin levels (TSH), thyroxine (T4), and 3,5,3’-triiodothyronine (T3) levels were recorded at baseline. We originally identified 1,629 patients in the 2013 baseline survey; however, owing to adjustments in the type of disease and definition in our study, the resulting total cohort included 1,284 individuals who were eligible for follow-up. Of these, 1,190 completed the follow-up study in 2019, including 549 from the Se-adequate area and 641 from the Se-deficient area (Fig. 1). A detailed description of the inclusion and exclusion criteria is provided in the Supplementary Materials (Table S1).

Based on previous findings, we estimated the recovery rate of thyroid function in patients with (sub-)clinical hypothyroidism residing in counties with different Se levels [21–23]. We determined that a sample size of 86 to 280 participants would be necessary to achieve a study power of 90% at an α level of 5%. However, given the possibility that individuals may become ineligible or withdraw from the study, our sample size target for patients with (sub-)clinical hypothyroidism was 350.

This study was conducted in accordance with the principles of the Declaration of Helsinki and was approved by the Medical Ethics Committee of Xi’an Jiaotong University (ethical approval file number: #2019–874). All participants provided written informed consent before enrollment in the study.

### Data collection

The personnel conducting the study received uniform training to ensure consistency in the field surveys. Semi-structured questionnaires were used to collect data on follow-up participants through face-to-face interviews. The questionnaire comprised 35 items including demographic information such as date of birth, sex, education, marital status, and occupation. Life behavior characteristics, such as smoking status, alcohol consumption, frequency of drinking tea, frequency of eating pickles, type of salt, and frequency of kelp/seaweed consumption, were also included. Additionally, disease and health conditions, such as history of thyroid disease, use of drugs affecting thyroid function, disease type, diagnosis time, and recovery status, were recorded. Physical examinations performed during the visits included assessments of height, weight, heart rate, thyroid palpation, exophthalmos, thyroid function tests, thyroid B-ultrasound, and additional analyses. Details are provided in the Supplementary Materials (Table S2).

### Sampling

Experienced nurses collected venous blood samples (5 mL) from a local county hospital to prepare serum. A subset of patients was randomly selected to provide additional samples of 5–10 mL of midstream urine and fingernail clippings (> 1 g), along with their serum samples. All specimens were frozen until shipment and analyses were performed.

### Laboratory analyses

Serum Se, nail Se, urinary *I*, and thyroid function indicators were measured. Serum Se levels were quantified using a dual-channel hydride generation atomic fluorescence photometer (AFS-2202E; Beijing Haiguang Instrument Co.). The same method was used to detect Se levels in the nail, water, rice, and wheat samples [20]. A standardized arsenic-cerium-catalyzed spectrophotometric assay was used to detect urinary *I* concentrations. Thyroid function was assessed using radioimmunoassays and chemiluminescence for serum TSH, T3, T4, and TPO-Ab, according to the manufacturer’s instructions (Weifang Sanwei Bioengineering Group Co., Ltd., Beijing North Institute of BIOTECHNOLOGY Co., Ltd., Siemens Healthcare Diagnostics Limited). The normal reference values were as follows: TPO-Ab: < 35 IU/mL (2013), < 15 U/mL (2019); TSH: 0.25–5 μIU/mL; T3: 0.78–2.20 ng/mL; T4: 4.2–13.5 μg/dL.

### Diagnostic criteria

Thyroid function recovery was defined as negative TPO-Ab levels and normal T3, T4, and TSH. Additionally, a cutoff value for TPO-Ab positivity was established in our laboratory using 300 healthy individuals not involved in the study, resulting in a value of 15 U/mL (radioimmunoassay). The limit of serum Se deficiency was set at 80 μg/L, which is consistent with our previous analyses [19]. The diagnostic criteria for thyroid disease are listed in the Supplementary Materials (Table S3).

### Statistical analysis

For continuous variables, the mean and standard deviation (X̅ ± SD) were used following a normal distribution. Otherwise, the median and interquartile range (IQR) were used. Categorical variables are reported as frequencies and percentages. Differences between groups were compared using the chi-square test and rank-sum test (Mann–Whitney U and Kruskal–Wallis tests). Differences between continuous variables with skewed distributions between the two groups were compared using the Mann–Whitney nonparametric test. The Wilcoxon signed-rank test was used to compare the differences in serum Se levels between baseline and follow-up. Multivariate regression analysis was conducted to explore the association between demographic characteristics, life behavior characteristics, and the recovery rate of thyroid function, as well as to estimate odds ratios (OR) and 95% confidence intervals (CI). We also used a restricted cubic spline (RCS) to explore the association between age at follow-up and significant recovery of thyroid function by sex. Statistical analysis was performed using SPSS version 18.0, GraphPad Prism v.8 (GraphPad Software Inc., San Diego, CA, USA), and R statistical software version 4.2.1 (package rms, ggplot2). Statistical significance was defined as a *P* value < 0.05 for two-tailed analysis.

## Results

### Participants and their demographic and lifestyle characteristics by county

The two groups did not significantly differ in terms of sex or smoking status. However, significant differences were observed for age, education level, body mass index (BMI), and alcohol consumption. Individuals from the Se-deficient county were younger on average than those from the Se-adequate county (median age: 56.0 vs. 61.0, Z = -5.530, *P* < 0.001). The level of education was generally higher in Se-deficient counties (*P* < 0.001). Additionally, participants from Se-deficient counties displayed a relatively high consumption of alcohol, with 24.5% drinking occasionally and 8.9% drinking frequently (Table 1).

**Table 1:** Demographic and lifestyle characteristics of the participants in the two counties

	Se-adequate		Se-deficient		*χ*^*2*^	*P*
	*N*(549)	%	*N*(641)	%		
**Age2019(years)**					10.872	0.012
18–30	3	0.5	11	1.7		
31–40	32	5.8	42	6.6		
41–50	72	13.1	118	18.4		
> = 51	442	80.5	470	73.3		
**Gender**					3.604	0.058
Male	122	22.2	173	27.0		
Female	427	77.8	468	73.0		
**Education**					46.777	< 0.001
Elementary school and below	438	79.8	395	61.6		
middle school	70	12.8	162	25.3		
High school / technical secondary school	24	4.4	52	8.1		
University and above	17	3.1	32	5.0		
**Smoking status**					2.380	0.304
Never	467	85.1	527	82.2		
Occasional smokers	15	2.7	16	2.5		
Frequent smokers	67	12.2	98	15.3		
**BMI**					25.944	< 0.001
< 18.5	29	5.3	47	7.3		
18.5 ~ 23.99	350	68.8	317	49.5		
24 ~ 27.99	144	26.2	222	34.6		
> = 28	26	4.7	55	8.6		
**Alcohol consumption**					13.862	0.001
Never	418	76.1	427	66.6		
Occasional	102	18.6	157	24.5		
Frequent	29	5.3	57	8.9		

During the follow-up investigation, we randomly selected participants from both counties to test nail Se levels and urinary *I* concentrations. The individuals from the Se-adequate county had a higher median Se content in their nails than those of the Se-deficient county (sample size: Se-adequate vs. Se-deficient: 47 vs. 74; 627.3 μg/kg vs. 358.0 μg/kg, Z = –8.381, *P* < 0.001); individuals from the Se-deficient county had higher urinary *I* concentrations (sample size: Se-deficient vs. Se-adequate: 81 vs. 66; 167.6 μg/L vs. 279.2 μg/L, Z = –4.187, *P* < 0.001). Information on the changes in serum Se levels between the two investigations is provided as Supplementary Materials (Figure S1).

### Recovery rate of thyroid function by county

After excluding the effect of drugs, thyroid function recovery in patients with subclinical hypothyroidism residing in Se-deficient areas was significantly better than that in Se-adequate areas (46.0% vs. 30.6%, *P* = 0.008), and recovery from all other diseases did not show significant differences between the two areas (Table 2).

**Table 2:** Recovery rates of thyroid function in various AITD subjects in areas with different Se levels

	Se-adequate			Se-deficient			*χ*^*2*^	*P*
	*N*	recovery	%	*N*	recovery	%		
Subclinical hyperthyroidism	17	8	47.1	9	4	44.4	-	0.613^a^
Hypothyroidism	12	6	50.0	30	9	30.0	0.749	0.387^b^
**Subclinical hypothyroidism**	98	30	**30.6**	285	131	**46.0**	7.054	**0.008**
HT + GD	36	3	8.3	29	2	6.9	0.047	0.829
Single TPO-Ab positive	73	19	26.0	86	28	32.6	0.809	0.368
**Total thyroid disorders**	236	66	**28.0**	439	174	**39.6**	9.122	**0.003**
Subjects with positive TPO-Ab at baseline	131	26	19.8	160	39	24.4	0.851	0.356

^a^The *P* value came from Fisher's Exact Test

^b^The *P* value came from Continuity Correction

We combined the limited number of individuals with clinical hypothyroidism with patients with subclinical hypothyroidism. We found that individuals residing in Se-deficient areas still had a higher rate of thyroid function recovery (44.4% vs. 32.7%, *χ*^*2*^ = 4.613, *P* = 0.032). We focused subsequent analysis of patients with subclinical and clinical hypothyroidism, collectively referred to as (sub-)clinical hypothyroidism, based on the recovery data of various disorders.

### Parameters affecting the recovery rate of thyroid function in (sub-)clinical hypothyroidism

This study included 425 participants with (sub-)clinical hypothyroidism. During the observation period, an exploratory analysis was performed to investigate the potential factors affecting recovery of thyroid function in patients with (sub-)clinical hypothyroidism. Variables significantly correlated with thyroid function recovery included place of residence (Se-deficient or Se-sufficient), age, sex, smoking status, and alcohol intake (Table 3). Participants who consumed alcohol in Se-deficient areas showed a higher rate of thyroid function recovery. Women exhibited a lower rate of recovery of thyroid function than men.

**Table 3:** Potential parameters affecting the recovery rate of thyroid function in (sub-)clinical hypothyroidism

	Recovery		Non-recovery		Recovery rate (%)	*χ*^*2*^* / Z*	*P*
	*N*(176)	%	*N*(249)	%			
**Location**						4.613	**0.032**
Se-adequate	36	20.5	74	29.7	32.7		
Se-deficient	140	79.5	175	70.3	44.4		
**Age2019**						2.904	0.407
18–30	5	2.8	4	1.6	55.6		
31–40	13	7.4	18	7.2	41.9		
41–50	39	22.2	42	16.9	48.1		
> = 51	119	67.6	185	74.3	39.1		
**Age (years)**						-2.707	0.007
**Serum Se at baseline**						-0.530	0.596
**Gender**						6.679	**0.010**
Male	69	39.2	68	27.3	50.4		
Female	107	60.8	181	72.7	37.2		
**Education**						2.163	0.539
Elementary school and below	104	59.1	164	65.9	38.9		
middle school	47	26.7	57	22.9	45.2		
High school / technical secondary school	17	9.7	20	8.0	46.0		
University and above	8	4.5	8	3.2	50.0		
**Smoking status**						12.723	**0.002**
Never	130	73.9	208	83.5	38.5		
Occasional smokers	3	1.7	11	4.4	21.4		
Frequent smokers	43	24.4	30	12.0	58.9		
**BMI**						6.685	0.083
< 18.5	9	5.1	26	10.4	28.1		
18.5 ~ 23.99	91	51.7	136	54.6	40.7		
24 ~ 27.99	62	35.2	65	26.1	48.7		
> = 28	14	8.0	22	8.8	40.6		
**Alcohol consumption**						10.548	**0.005**
Never	99	56.3	178	71.5	35.7		
Occasional	54	30.7	50	20.1	51.9		
Frequent	23	13.1	21	8.4	52.3		

### Multivariate analysis of parameters affecting the recovery rate of thyroid function in (sub-)clinical hypothyroidism

Based on the binary logistic regression analysis, female sex and increasing age were identified as factors that hindered recovery of thyroid function (Table 4). The area of residence was significant in the univariate analysis. However, multifactor analysis appeared to exclude this effect, and BMI appeared to be unrelated to the recovery of thyroid function.

**Table 4:** Logistic regression analysis of parameters affecting thyroid function recovery^a^

Variable		model 1			model 2^b^		
		OR	OR 95%CI	*P*	OR	OR 95%CI	*P*
Location	Se-adequate	1			1		
	Se-deficient	0.626	0.365–1.073	0.088	0.634	0.366–1.098	0.104
Gender	Male	1			1		
	Female	1.977	1.279–3.055	**0.002**	1.875	1.080–3.257	**0.026**
Age		1.027	1.007–1.047	**0.007**	1.028	1.007–1.049	**0.009**
BMI	18.5 ~ 23.99	1			1		
	< 18.5	1.934	0.850–4.400	0.116	1.958	0.842–4.556	0.116
	24 ~ 27.99	0.709	0.450–1.115	0.137	0.693	0.437–1.100	0.137
	> = 28	1.068	0.507–2.249	0.863	1.031	0.486–2.186	0.863
Serum Se at baseline		0.997	0.991–1.004	0.404	0.997	0.991–1.003	0.374

^a^Dependent variable assignment: Thyroid function turns recovery -1, others -2

^b^Adjusted model 2 included 3 additional confounders (smoking, alcohol intake, and education). The boldface denotes statistical significance

**Fig. 2:**
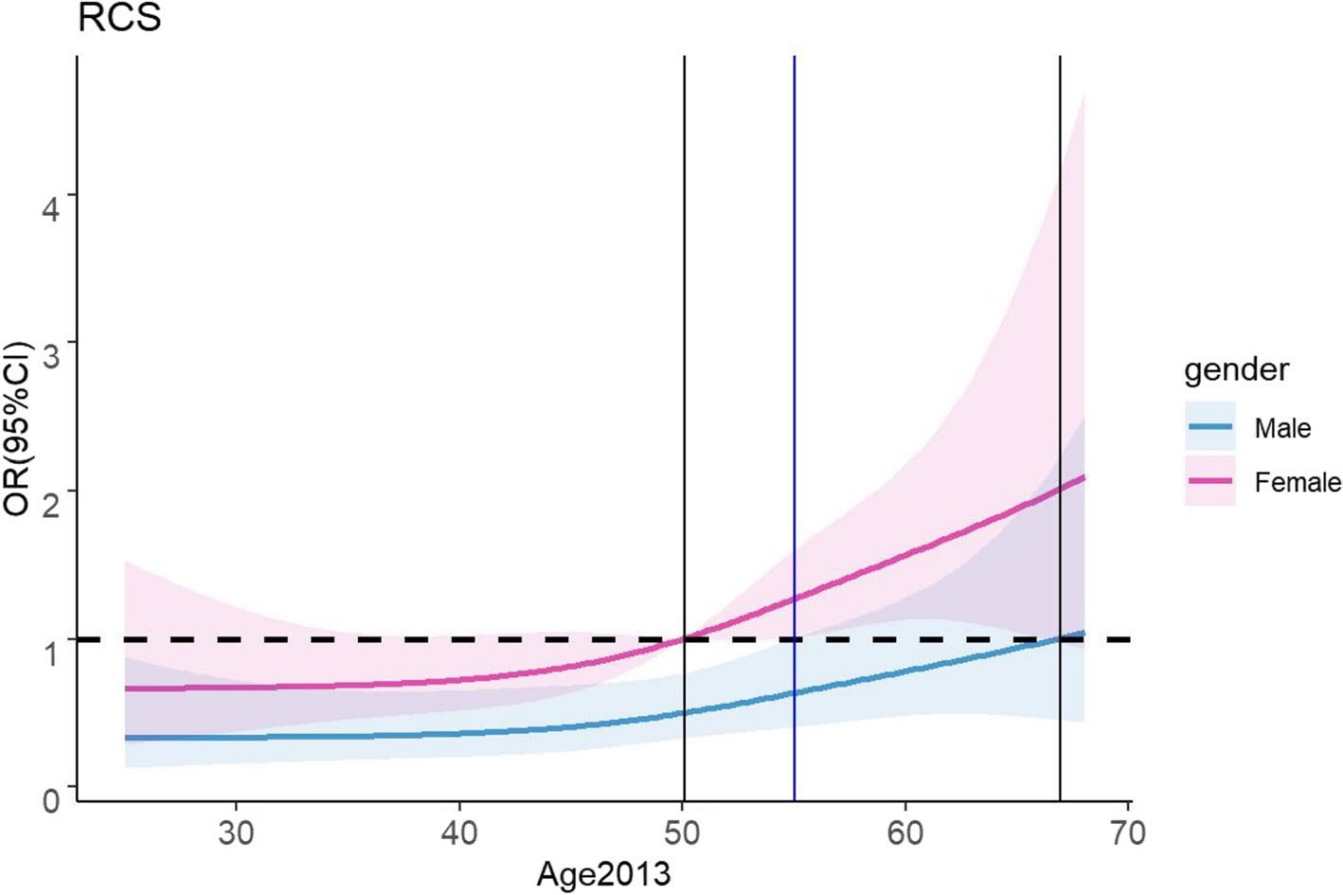
Association between baseline age and significant thyroid function recovery by sex

We conducted an in-depth analysis to determine how sex and age at follow-up affected research outcomes (*P*_overall_ = 0.0019). By stratifying the results by sex, we found that recovery of thyroid function varied with age in individuals of different sexes, with females exhibiting an OR of 1 at 51 years (beyond which the OR significantly increased with age) and males showing the same trend at 67 years. When we focused on OR95%CI, we observed a threshold of 55 years old. In those less than 55 years old, male sex showed a protective effect, while in those over 55 years old, female sex showed a risk effect (Fig. 2).

## Discussion

### Complex interrelationship between Se status and hypothyroidism

Our study was a large longitudinal cohort study spanning six years, addressing the rarely explored environmental factors affecting the recovery of thyroid function in individuals with (sub-)clinical hypothyroidism. The relatively long follow-up duration and follow-up rate reinforces our analysis and improves the reliability of our findings. The Se concentrations in the nail and serum samples of the participants supported the selection of the study areas and revealed an Se status almost two times higher in the Se-adequate county of Ziyang than in the Se-deficient county of Ningshan, which was consistent with the baseline survey [19]. Notably, the recovery rate for thyroid dysfunction, particularly (sub-)clinical hypothyroidism, is higher in Se-deficient counties (Ningshan).

The effects of Se supplementation in patients with thyroid disease may differ based on the baseline levels [12, 13]. Several interventional studies have reported no health benefits from Se intake or any effect on thyroid gland appearance or thyroid autoantibody titers [10, 11, 24]. Nonetheless, some Se supplementation trials have reported positive effects on thyroid appearance during ultrasound and autoantibody titers. For instance, a seminal randomized control study using 200 µg of sodium selenite per day for 3 months [25] or later studies testing a similar regimen with varying dosages of selenite or selenomethionine over different periods [26]. Notably, most of these studies were conducted in areas with low Se supply, where supplemental Se served as a substitution therapy rather than pharmacological. The underlying mechanism likely involves increased biosynthesis of selenoproteins [27, 28]. Some studies have highlighted the central role of glutathione peroxidase 4 (GPX4) in preventing ferroptosis of autoimmune-relevant neutrophils [29] and follicular helper T cells to mount antibody responses to infection and vaccination [30], which have supported the general relevance of selenoproteins in autoimmune disease development and immune system function. Therefore, severe Se deficiency is a relevant and preventable risk factor for the development of autoantibodies and autoimmune disease [31]. This is supported by a randomized controlled study that showed a significant reduction in postpartum thyroiditis when supplemental Se was administered to healthy pregnant women who were positive for TPO-Ab [32]. The design of this study is not perfectly compatible with the reported prospective cohort study but highlights the relevant role of Se deficiency in thyroid autoimmunity. Our findings showed that TPOAb-positive patients had a higher rate of thyroid function recovery in Se-deficient areas (24.4% vs. 18.9%); however, this difference was not significant. Collectively, these findings emphasize the complex but significant relationship between Se status and thyroid disease.

The significantly higher recovery rate of thyroid function in patients with (sub-)clinical hypothyroidism in the Se-deficient area may be related to the tendency for better Se status in the enrolled participants, and there is a possibility of sex bias in Se-deficient areas, with a higher proportion of males residing in these regions. Our study found that the higher recovery rate of thyroid function in Se-deficient areas may be attributable to increased population mobility, higher living standards, and reduced Se shadowing due to geographic restrictions. Additionally, compared to Se-adequate areas, there is a clearer increasing trend in Se-deficient areas (especially in the patients with sub-clinical hypothyroidism). Serum Se levels of the population in Se-rich areas were higher than 80 ug/L (i.e., in a Se-sufficient state) at both baseline and follow-up (Figure S1), and they suffered from hypothyroidism, which may not be directly related to Se. In contrast, the development of hypothyroidism in populations living in Se-deficient areas may have a higher Se correlation. The thyroid gland actively accumulates Se, and has the highest Se content in the human body. Hence, even a subtle increase in serum Se levels could be associated with better Se status in the thyroid gland and immune cells. Although this theory is yet to be tested in humans, our longitudinal analysis of thyroid hormone changes suggests that improved Se status supports recovery from thyroid disease. For example, the TSH level improved to normal in 21.1% of individuals residing in Se-deficient counties compared to –3.2% in Se-adequate areas, while the T3 level improved in 14.1% of individuals residing in Se-deficient counties compared to 13.8% in Se-adequate counties, and the T4 level improved in 7.5% of individuals residing in Se-deficient counties compared to 5.1% in Se-adequate counties (Table S2). These findings further support the interpretation that an improved Se status supports recovery from thyroid diseases.

### Other factors affecting thyroid function

*I* plays a crucial role in thyroid hormone synthesis and insufficient or excessive intake can negatively impact health [33–37]. We found that the urinary *I* level in Se-deficient counties was significantly higher than that in Se-adequate counties; however, both groups were *I*-sufficient. Previous studies have indicated that the *I* enhancement policy in China has helped achieve the lowest prevalence of thyroid-related diseases when the median urinary *I* concentration ranged between 100 and 300 μg/L [38], which was the case for both groups in our study [39]. Therefore, this policy likely improved *I* intake in both counties and did not appear to affect the outcome of our study.

In our final multivariate analysis, age and sex were related to the rate of thyroid function recovery, consistent with previous studies [40–42]. As shown in Fig. 2, there was a significant sex difference at 55 years, with men under 55 years acting as a protective factor (OR < 1) and women aged > 55 years acting as a risk factor (OR > 1), which might be linked to changes in human sex hormones. Previous studies reported that the prevalence of thyroid diseases increases in postmenopausal women [43]. Furthermore, a study stratified by menopause exploring the relationship between polybrominated diphenyl ethers (PBDEs) (an environmental toxicant that disrupts thyroid hormones and estrogenic activity) and thyroid disease in women suggested that altered estrogen levels during menopause may enhance the disruption of thyroid signaling by PBDEs [44]. These findings demonstrate that changes in sex hormone levels can influence thyroid function.

### Limitations

The observational design of this study did not allow for causal inferences. We cannot ensure that the same group of observed participants is always at the same exposure level, which may affect the outcomes. At the same time, due to financial constraints, we were unable to detect urinary I concentrations in all participants, which may have led to some bias.

Moreover, we made some adjustments to the type of disease and study population definition to include more patients, resulting in losses to follow-up. Therefore, there were some data differences between this study and the baseline cross-sectional study [19]. Nevertheless, it is essential to note that these limitations do not diminish the significance of our findings.

## Conclusions

Our six-year follow-up study revealed that female sex and increasing age in individuals over 30 years of age negatively affect recovery of thyroid function in patients with (sub-)clinical hypothyroidism, with menopause potentially being an essential period. However, Se did not significantly affect the recovery of patients with (sub-)clinical hypothyroidism, indicating that the complex manifestations of Se require further exploratory research.

### Supplementary Information

**Additional file 1: Table S1. **Inclusion and Exclusion Criteria. **Table S2. **Comparison of the change of thyroid function in different investigating times by county. **Table S3.** Diagnostic criteria for thyroid diseases. **Figure S1. **Changes in serum selenium levels in the whole group (A) and patients with (sub-)clinical hypothyroidism (H) in areas with different selenium levels during a 6-year observation period.

## Data Availability

The datasets generated and/or analyzed during the current study are not publicly available, but are available from the corresponding author on reasonable request.

## Abbreviations

AITD: Autoimmune thyroid disease
GD: Graves' disease
AIT: Autoimmune thyroiditis
TPO-Ab: Thyroid peroxidase antibody
HT: Hashimoto thyroiditis
TSH: Thyrotropin levels
T4: Thyroxine
T3: 3,5,3′-Triiodothyronine
GPX4: Glutathione peroxidase 4
PBDEs: Polybrominated diphenyl ethers
Se: Selenium
*I*: Iodine
OR: Odds ratio
CI: Confidence interval
RCS: Restricted cubic spline
