# Supplementary material for: A follow-up study on factors affecting the recovery of patients with hypothyroidism in different selenium environments
Source: BMC Endocr Disord. 2024 Jan 29;24:14. doi: 10.1186/s12902-024-01536-1 (PMC10823702; doi:10.1186/s12902-024-01536-1)
Supplement: Supplementary file 1 — Additional file 1: Table S1. Inclusion and Exclusion Criteria. Table S2. Comparison of the change of thyroid function in different investigating times by county. Table S3. Diagnostic criteria for thyroid diseases. Figure S1. Changes in serum selenium levels in the whole group (A) and patients with (sub-)clinical hypothyroidism (H) in areas with different selenium levels during a 6-year observation period. [file 12902_2024_1536_MOESM1_ESM.docx]

## Appendices

**Table S1:** Inclusion and Exclusion Criteria

| **Inclusion and Exclusion Criteria：** |
| --- |
| **Inclusion criteria：** |
| a) The population who have been detected with serum selenium content, thyroid peroxidase antibody (TPO-Ab), thyrotropin (TSH), thyroxine (T_4_), and triiodothyronine (T_3_) at baseline survey. |
| b) Population with complete information on covariates such as educational level, marriage, occupation, smoking status, alcohol consumption, eating habits, and so on. |
| c) Those who have self-reported or diagnosed with thyroid-related disease or been in the subclinical stage at baseline. |
| d)Those who participate in the follow-up survey voluntarily and have signed the informed consent. |
| e) The participants who can cooperate in completing the questionnaire, and physical and blood examinations. |
| f) Those who have not moved out of the local area in the past 6 years or out for less than 1 year. |
| **Exclusion criteria:** |
| a) Patients with autoimmune diseases (such as Systemic Lupus Erythematosus, type 1 diabetes, etc.), women with abnormal thyroid function during pregnancy, those with renal insufficiency, liver insufficiency, severe systemic diseases, or chronic wasting diseases. |
| b)Those who have taken *I*-containing drugs or contrast agents within the past 6 months; |
| c)Women under the age of 18 or pregnant. |
| The research protocol was approved by the Medical Ethics Committee of Xi’an Jiaotong University. The ethical approval file number was 2019-874. All participants provided written informed consent before participating in the study. |

**Table S2:** Comparison of the change of thyroid function in different investigating times by county

| County | Index | | Baseline | | Follow-up | | Δ% | Z | *P* |
| --- | --- | --- | --- | --- | --- | --- | --- | --- | --- |
|  |  |  | N | % | N | % |  |  |  |
| Se-adequate | TPO-Ab | Normal | 371 | 67.6 | 401 | 73.2 | 5.5 | -3.444 | 0.001 |
|  |  | High | 178 | 32.4 | 147 | 26.8 |  |  |  |
|  | TSH | Low | 28 | 5.1 | 12 | 2.2 |  | -3.800 | <0.001 |
|  |  | Normal | 347 | 63.2 | 330 | 60.2 | -3.2 |  |  |
|  |  | High | 174 | 31.7 | 206 | 37.6 |  |  |  |
|  | T_3_ | Low | 116 | 21.1 | 25 | 4.6 |  | -8.223 | <0.001 |
|  |  | Normal | 419 | 76.3 | 494 | 90.1 | 13.8 |  |  |
|  |  | High | 14 | 2.6 | 29 | 5.3 |  |  |  |
|  | T_4_ | Low | 40 | 7.3 | 14 | 2.6 |  | -2.650 | 0.008 |
|  |  | Normal | 479 | 87.2 | 506 | 92.3 | 5.1 |  |  |
|  |  | High | 30 | 5.5 | 28 | 5.1 |  |  |  |
|  |  |  |  |  |  |  |  |  |  |
| Se-deficient | TPO-Ab | Normal | 440 | 68.6 | 479 | 75.3 | 6.7 | -3.697 | <0.001 |
|  |  | High | 201 | 31.4 | 157 | 24.7 |  |  |  |
|  | TSH | Low | 13 | 2.0 | 9 | 1.4 |  | -7.019 | <0.001 |
|  |  | Normal | 232 | 36.2 | 365 | 57.3 | 21.1 |  |  |
|  |  | High | 396 | 61.8 | 263 | 41.3 |  |  |  |
|  | T_3_ | Low | 115 | 17.9 | 48 | 7.5 |  | -3.660 | <0.001 |
|  |  | Normal | 487 | 76.0 | 574 | 90.1 | 14.1 |  |  |
|  |  | High | 39 | 6.1 | 15 | 2.4 |  |  |  |
|  | T_4_ | Low | 55 | 8.6 | 12 | 1.9 |  | -3.093 | 0.002 |
|  |  | Normal | 538 | 83.9 | 582 | 91.4 | 7.5 |  |  |
|  |  | High | 48 | 7.5 | 43 | 6.8 |  |  |  |

**Table S3:** Diagnostic criteria for thyroid diseases

| **Thyroid condition** | **Diagnostic Criteria** |
| --- | --- |
| Subclinical hypothyroidism | TSH > 5.0μIU/mL and 13.5μg/dL ≥ T_4_ ≥ 4.2μg/dL |
| Overt hypothyroidism | TSH > 5.0μIU/mL and T_4_ < 4.2μg/dL, or history of overt hypothyroidism |
| Subclinical hyperthyroidism | TSH < 0.25μIU/mL, 13.5μg/dL ≥ T_4_ ≥ 4.2μg/dL |
| Hashimoto thyroiditis (HT) | 1. TPOAb positive. 2. Normal thyroid function, clinical or subclinical hypothyroidism. 3. Diffuse enlargement of the thyroid gland by ultrasound. 4. Echogenicity within the thyroid gland by ultrasound. |
| Graves’ disease (GD) | 1. Obvious signs and symptoms of hyperthyroidism. 2. Diffuse enlargement of the thyroid gland detected by ultrasound or palpation. 3. Decreased TSH and elevated T4. 4. Ocular proptosis and other infiltrative eye signs. 5. Anterior tibial fluid edema. 6. TPOAb positive, or TRAb^a^ > 2IU/L. (1-3 are diagnostic prerequisites, 4~6 are auxiliary conditions.) |
| Single TPO-Ab positive | Only TPOAb positive. |

^a^ TRAb: thyrotropin (TSH) receptor antibodies.


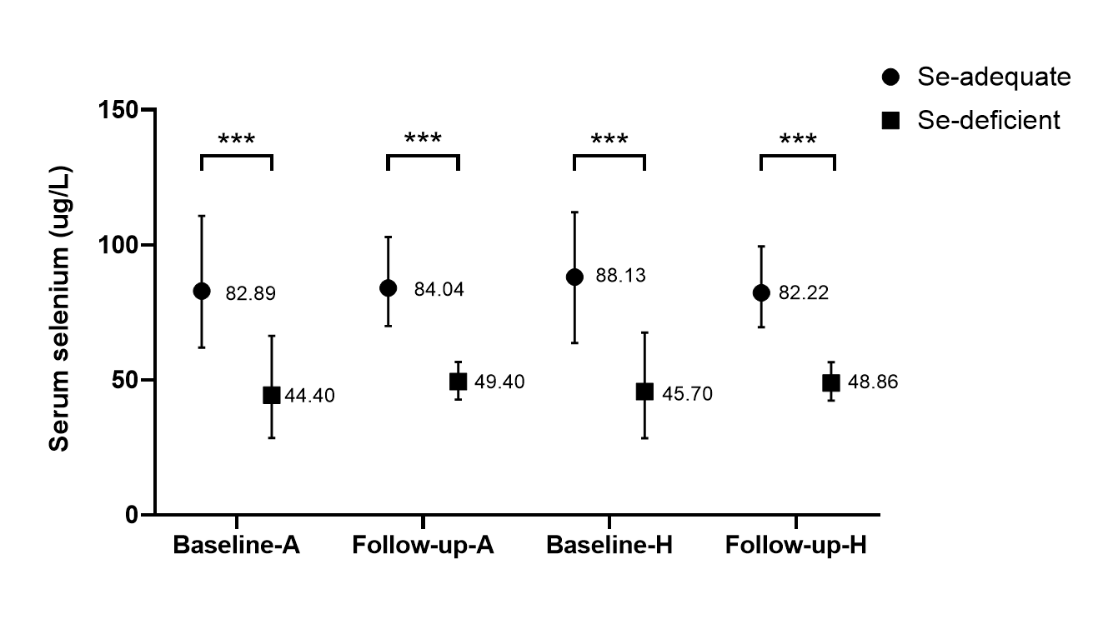


**Figure S1:** Changes in serum selenium levels in the whole group (A) and patients with (sub-)clinical hypothyroidism (H) in areas with different selenium levels during a 6-year observation period.

Serum selenium was significantly different between the two regions of patients at all cross-sectional times (*P*<0.001). However, there was no significant difference in time longitudinal for patients in each zone. A: Change of serum selenium in whole subjects (Se-adequate: Z=-1.512, *P*=0.131; Se-deficient: Z=-1.636, *P*=0.102). H: Change of serum selenium in (sub-) clinical hypothyroidism (Se-adequate: Z=-0.388, *P*=0.698; Se-deficient: Z=-0.479, *P*=0.632).
